# Appropriateness of digital vending machines and mobile phone applications in increasing access to HIV testing among adolescents and young people aged 18–24 in an urban community in Zambia: insights from adolescents, young people and healthcare providers

**DOI:** 10.1080/16549716.2026.2702213

**Published:** 2026-09-23

**Authors:** Sarah Mukimba, Joseph Mumba Zulu, Choolwe Jacobs, Patricia Maritim, Jaime H. Vera, Mary Darking, Adam Silumbwe, Musonda Simwinga

**Affiliations:** a Department of Health Promotion and Education, School of Public Health, University of Zambia, Lusaka, Zambia; b Department of Epidemiology, School of Public Health, University of Zambia, Lusaka, Zambia; c Department of Health Policy, School of Public Health, University of Zambia, Lusaka, Zambia; d Department of Global Health and Infection, Brighton and Sussex Medical School, University of Sussex, Brighton, UK; e School of Applied Social Science, University of Brighton, Brighton, UK; f Department of Social Science, Zambart, Lusaka, Zambia

**Keywords:** HIV self-testing, digital health, implementation science, diffusion of innovations, sub-Saharan Africa

## Abstract

**Background:**

Despite several initiatives aimed at increasing HIV testing among young people in Zambia, testing uptake remains low, highlighting the need for innovative approaches.

**Objective:**

This paper aimed to explore perceptions of the appropriateness of a proposed digital HIV testing pathway, comprising digital vending machines and a mobile phone application for improving access to HIV self-testing among adolescents and young people in Lusaka, Zambia.

**Methods:**

This was a formative qualitative study conducted in an urban community of Lusaka district. Purposive sampling was used to recruit adolescents, young people and healthcare providers. Participants were invited to reflect on a conceptual description of a proposed digital HIV testing pathway during in-depth interviews and focus group discussions. Data were analysed thematically, guided by Rogers’ diffusion of innovations.

**Results:**

Participants perceived the concept of using digital vending machines as convenient and capable of facilitating quick access to HIV self-testing kits while promoting a culture of testing. However, concerns were raised regarding the need for emotional support following a positive result. The accompanying mobile phone application was perceived as potentially appropriate for linkage to emotional support although barriers related to smartphone access, digital literacy and usability were highlighted.

**Conclusion:**

Our study suggests that digital vending machines and mobile phone applications may offer a promising approach to expanding HIV testing among adolescents and young people. However, considerations related to digital access, privacy, user-friendly design and strengthened linkage to care mechanisms are critical to enhance their potential appropriateness in this context.

## Paper Context

- **Main findings:** Digital vending machines and mobile phone applications were perceived as potentially improving access to human immunodeficiency virus testing among young people through increased convenience, privacy and timely access to self-test kits and emotional support.
- **Added knowledge:** This study provides formative evidence on how young people and healthcare providers perceive a proposed digital-supported testing pathway, highlighting both perceived benefits and concerns related to usability, stigma and linkage to care.
- **Global health impact for policy and action:** Findings suggest that while digital approaches may enhance access to testing, careful attention to privacy, system integration and post-test support is needed before implementation in similar low-resource settings.

## Background

In 2023, around 360,000 young people aged 15–24 years globally were newly infected with HIV [1]. In the African region, especially sub-Saharan Africa, young people aged between 15 and 24 years accounted for the highest number of HIV cases worldwide, with 1.9 million young women and 1.2 million young men living with HIV [2]. In Zambia, about 6.6% of young people aged 15–24 years are living with HIV, with this age group accounting for 9.4% of all people living with HIV (PLHIV) [3]. Adolescents and young people (AYP) experience a high burden of new HIV infections and represent an important population for targeted HIV prevention and testing interventions [4].

The expansion of HIV testing coverage is crucial for achieving one of the UNAIDS 95–95–95 target of ensuring that 95% of PLHIV are aware of their status [5]. Attaining this target has, however, been slow, particularly among the AYP, with only about 87% of this population aware of their HIV status globally [1]. In Zambia, only 42% of adolescents have ever tested for HIV according to the Zambia Demographic Health Survey [6]. One of the key challenges is that AYP are often hesitant to get tested for HIV despite awareness of where such services are offered because of confidentiality and convenience concerns. Moreover, local health services generally tend not to be adolescent friendly or responsive due to factors, such as judgement associated with seeking an HIV test [7].

In Zambia, initiatives to bring HIV testing services closer to young people, such as mobile clinics and home-based testing, have been implemented [8]. However, it has been noted in previous studies that AYP do not like to be counselled and tested through these methods as the service providers may personally recognize them from within the community [9–11]. Existing research in sexual and reproductive health has shown that digital technologies, such as vending machines, are largely acceptable and effective to overcome barriers to HIV testing among AYP because they offer increased convenience, privacy and confidentiality [12]. In this study, we explored participant perspectives regarding a proposed digital HIV testing pathway comprising a digital vending machine (DVM) for dispensing HIV self-test kits and an associated mobile phone application to support users after self-testing.

DVMs operate similarly to standard vending machines, allowing users to select and dispense items through a simple interface. In this study, we conceptualized the DVM as a platform for dispensing HIV self-test kits. After selecting a test kit option, users would then request and collect a sealed HIV self-test kit from the machine. The HIV self-test kits are designed for individual use, enabling users to collect their own sample (e.g. oral fluid of finger-prick blood sample) and perform the test independently by following the instructions provided in the kit. Users are also responsible for visually interpreting their results, which are typically available within a short period. These kits are pre-qualified rapid diagnostic tests with established sensitivity and specificity as recommended for HIV self-testing [13].

To complement the DVMs, a mobile phone application (Figure 2) supports users after self-testing. The application guides users on how to interpret their results, provides simplified explanations and offers recommendations for the next steps, including linkage to confirmatory testing and care where necessary. In this way, the mobile application functions as a supportive tool that works in concert with the DVM by enhancing result interpretation, maintaining confidentiality and facilitating linkage to care.

Placed in areas where AYP mostly frequent, such as schools and clinics, DVMs can help reduce stigma and barriers to healthcare access, by providing easy and discreet access to essential health products like HIV self-test kits [6]. However, limited research on DVMs for HIV testing exists and it largely focuses on adult men who have sex with men [8,9,14,15]. Furthermore, little is known about how such technologies are perceived by adolescents and young people in African settings.

Therefore, this paper explores the appropriateness of using the DVMs for accessing HIV self-test kits and a mobile phone application for accessing information and support among AYP aged 18–24 years in an urban community in Lusaka district, Zambia. According to Proctor et al. [16], appropriateness is ‘the perceived fit, relevance, or compatibility of the innovation or evidence-based practice for a given practice setting, provider, or consumer; and/or perceived fit of the innovation to address a particular issue or problem’. Consistent with principles of implementation research, appropriateness can be explored prospectively by assessing stakeholders’ perceptions prior to implementation. We operationalized appropriateness through participants’ anticipated perceptions of key innovation attributes, including perceived relative advantage, compatibility with existing values and experiences, and perceived complexity of use. These attributes are consistent with Rogers’ (1962) diffusion of innovations theory which highlights that adoption of innovations is influenced by such factors [17].

This study was part of a broader study at Zambart which aimed to co-design a digitally supported care pathway with young people to facilitate access to comprehensive sexual reproductive health services including HIV testing and treatment.

## Methods

### Theoretical framework

This study was guided by Rogers’ (1962) diffusion of innovations (DOI) theory [18]. This theory provided a lens through which we explored the perceived *innovation attributes*, the *relative advantage, the compatibility, and the complexity*, factors that would potentially influence adoption of the proposed digital HIV testing pathway within any social system [17]. These constructs of the DOI theory are explained in detail in Table 1. The development of the interview guides, data analysis and interpretation of findings were guided by the DOI theory. Although this theory has been widely applied in health research, its application to HIV self-testing delivery through DVMs and a mobile phone application among AYP in low-resource settings remains limited.

**Table 1.** Definitions of the characteristics of the innovation.

*Relative advantage*	The degree to which accessing an HIV test kit via a vending machine or receiving an HIV test result through a mobile phone app is perceived as being better than the idea of accessing both services through traditional methods.
*Compatibility*	The degree to which accessing an HIV test kit via a vending machine or receiving an HIV test result through a mobile phone app an innovation is perceived as consistent with the previous experience of accessing HIV tests via a range of different facilities and hubs, values and needs of potential adopters.
*Complexity*	The degree to which the vending machine and the mobile app is perceived as relatively difficult to understand and use.

## Study design

Prior to data collection, we explained to the selected participants about the proposed digital HIV testing pathway – the DVMs and a mobile phone application. Participants were shown Illustrative images (Figures 1 and 2) to facilitate understanding. Participants were also encouraged to ask questions to ensure a shared understanding of the proposed digital HIV testing pathway before expressing their views. We subsequently conducted a *formative qualitative* study to understand their perceptions, needs and contextual dynamics to inform the co-design process and implementation of the proposed interventions and identifying anticipated challenges and adaptations. This approach enabled us to explore the perceived fit, relevance and acceptability of the proposed innovations prior to full-scale deployment, thereby informing evidence-based implementation strategies to increase adoption. We followed the consolidated criteria for reporting qualitative research (COREQ) 32 item checklist which guided the design and reporting of the qualitative data [19].

**Figure 1.**
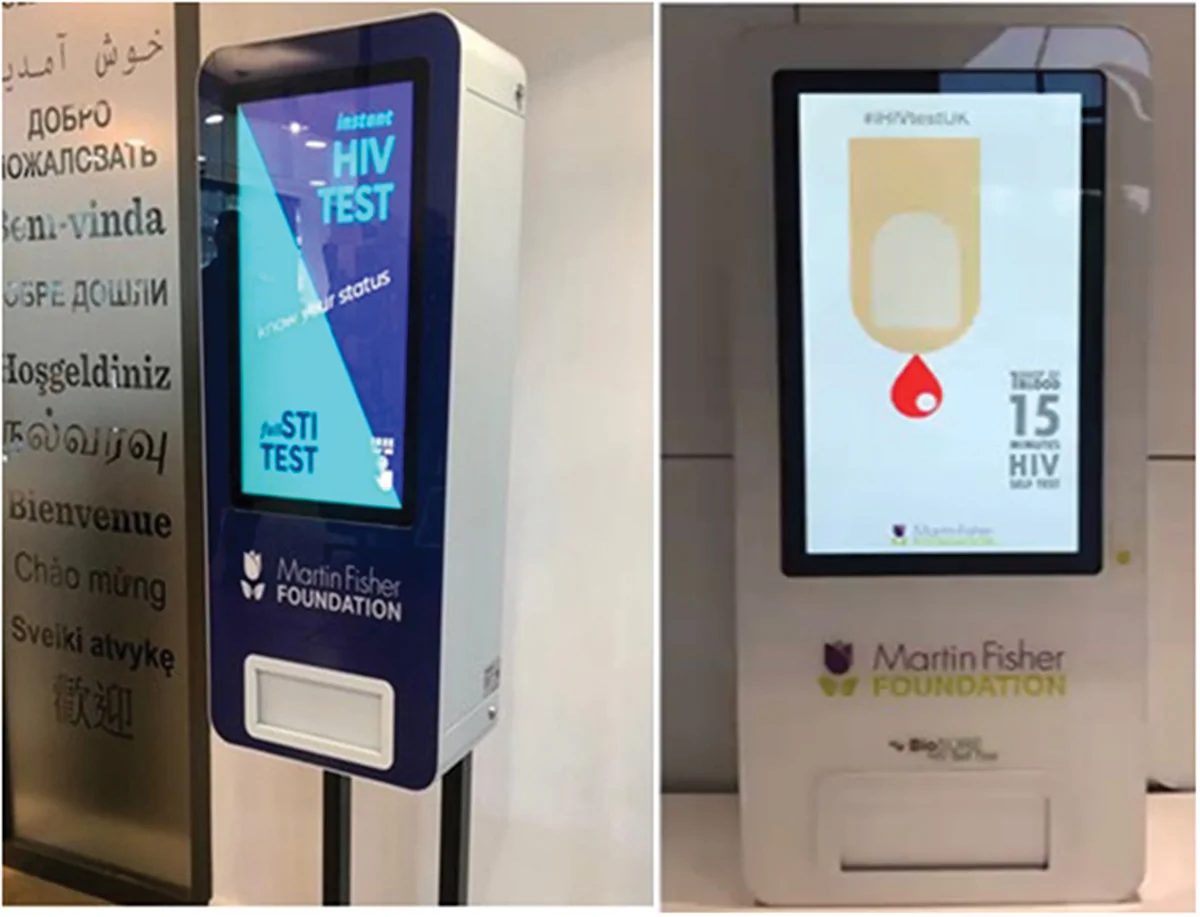
Proposed digital vending machine (DVM) for HIV self-testing services among adolescents and young people.

**Figure 2.**
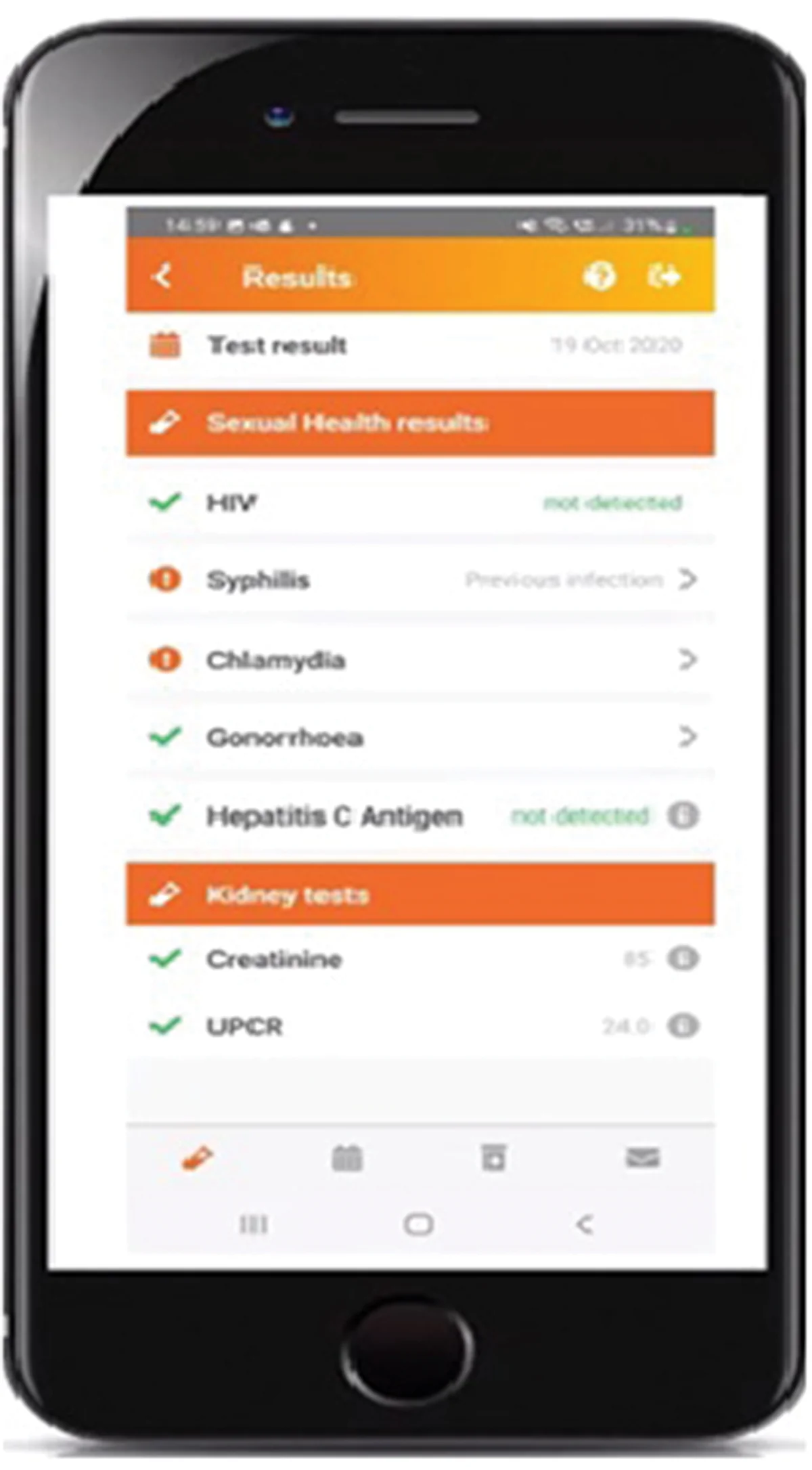
Proposed mobile phone application interface to support users after self-testing.

## Study setting

This study was conducted in a densely populated urban community in Lusaka, Zambia. The community has an estimated population of 40,000–50,000 people with relatively large household sizes averaging six members [20]. The community was selected due to its prior involvement in youth-focused HIV research and implementation initiatives, making it a relevant setting for exploration of youth-related health challenges [8].

## Sampling and recruitment of participants

Participants included adolescents and young people aged 18–24 years, peer support workers and healthcare workers (Table 2). Three eligible participants declined participation due to competing commitments. We used purposive sampling to recruit participants with prior experience accessing HIV testing services to enable reflection on existing testing pathways in comparison with the proposed intervention. Further, participants were purposively selected to ensure variation in gender and experience in utilization of either peer-led community-based hubs, youth-friendly corners or traditional healthcare facilities for HIV testing. The healthcare providers were selected based on their involvement in the provision of HIV services at their facilities. Recruitment of participants was facilitated by a community mobilizer familiar with the local context. There was no relationship with the participants prior to the commencement of this study.

**Table 2.** Study participants.

Category	Participants	Number
FGD		
	Men (15–24)	17
	Women (15–24)	16
IDI		
	Healthcare providers	4
	Peer support workers	4
Total		**41**

## Focus group discussions (FGDs)

Four FGDs were conducted with AYP (two male and two female groups), each comprising 8–9 participants. Gender-specific groups were used to promote open discussion since topics related to health and HIV testing are usually sensitive. Participants discussed their experiences with HIV testing and reflected on the perceived suitability of the proposed DVM and mobile application for delivering HIV self-testing services. In total, 33 AYP participated in the FGDs.

## In-depth interviews (IDIs) and semi-structured interviews

Four IDIs were conducted with peer support workers and another four IDIs were done with healthcare providers involved in HIV testing and counselling services. The interviews were held in private spaces to ensure privacy and confidentiality. Healthcare providers without prior experience in delivering HIV testing or counselling services to young people were not interviewed.

Three semi-structured interview guides were developed to facilitate conversations with AYP, peer support workers and healthcare providers. The guides were then piloted in a different community to ensure that the questions were correctly phrased to capture the different dimensions of the DOI theory.

## Data collection

Data were collected using face-to-face interviews conducted between April and June 2023 in English with the healthcare providers, while Bemba and Nyanja were used for the AYP and the peer supporters. All interviews were audio-recorded and transcribed verbatim, and those done in local languages were translated into English. The interviews lasted less than 2 h and were conducted by the first author, a trained female qualitative researcher. Field notes were taken to complement the recordings. Data collection continued until saturation was reached, which means we stopped collecting data at the point when no new information was observed from the additional interviews.

## Data analysis

Data were analysed manually using thematic analysis, an approach which entails searching across all the transcripts to identify, analyse and report repeated patterns in the text [21]. We deductively developed a coding structure based on the DOI’s three constructs, which we categorised as the main themes (Table 1). The sub-themes were inductively developed from a thorough reading of the transcripts and iterative discussions between the first author and the rest of the research team. Transcripts were read line-by-line to identify codes that were grouped into themes which were then reviewed collaboratively with all the co-authors and refined/rearranged to ensure alignment with study objectives. Themes were developed both within and across participant groups, allowing for an integrated understanding of users and provider perspectives. The findings were supported with verbatim quotes.

## Ethical consideration

Ethical approval was obtained from the University of Zambia Biomedical Research Ethics Committee (UNZABREC), Reference No. 3460-2022, National Health Research Authority, and Reference No: NHRA00006/08/02/2023. Participants were given an information sheet that detailed the objectives and potential risks and benefits of participating in this study. We made it clear to the participants that their participation was voluntary, and should they feel uncomfortable with the interviews, they were free to withdraw. After this, written informed consent was then obtained from the participants. During the interviews, we used participants, identifiers and not individual names to ensure anonymity in the data. This study was performed in accordance with the Declaration of Helsinki guidelines [22].

## Results

The data detailing the perspectives of adolescents, young people and healthcare providers are organized according to key constructs of the adapted DOI theory specifically the relative advantage, compatibility, and complexity (Table 3). While the results are organized within these domains, some themes, such as privacy, accessibility and stigma cut across multiple constructs, reflecting the interconnected nature of the proposed digital HIV testing pathway attributes. In such cases, themes were categorized based on their primary conceptual relevance. As the proposed DVMs and mobile phone application had not yet been implemented, participants’ responses reflect anticipated experiences and expectations, rather than direct interaction with the proposed digital HIV testing pathway.

**Table 3.** Major themes and sub-themes.

Construct	Main themes	Sub-themes
Relative advantage		
	Relative advantage of the digital vending machine	Convenience and accessibility
		Reduced stigma and judgement
		Faster access to HIV self-test Kits
		Promotion of routine testing
	Relative advantage of the mobile phone application	Timely support and information on linkage to care
Complexity		
	Complexity of using the digital vending machine	User interface and navigation
		Familiarity with vending technology
	Complexity of using the mobile phone application	Literacy and interpretation of results
Compatibility		
	Compatibility of the digital vending machine	Safety and security concerns
		Perceived privacy benefits
		Integration with existing healthcare systems
		Maintenance and Technical reliability
		Linkage to Care and emotional support
	Compatibility of the mobile phone application	Technological accessibility
		Familiarity with mobile technology
		Internet cost considerations

## Relative advantage

### Relative advantage of the digital vending machine

#### Convenience and accessibility

The AYP and healthcare workers perceived the digital vending machines as offering greater convenience compared to traditional HIV testing methods, highlighting its potential to increase access through multiple community locations and flexible availability, allowing young people to obtain HIV self-test kits (HIVST) without visiting health facilities. Healthcare workers further noted that community placement could improve access for adolescents facing mobility and structural barriers.

This thing is ok because it will not be at one place and there will be many and you will not find many people so you will go there at any time. [Male FGD participant]

They rarely come here to access HIV services … but if you put it near the market, meaning the adolescent will be able to come back from home and will access the service and go back home. [Healthcare worker, individual interview]

These findings suggest that the perceived relative advantage of the DVM is largely driven by improved accessibility and convenience, suggesting that decentralizing HIV services may help address structural and geographical barriers associated with facility-based testing.

#### Reduced stigma and judgement

The participants described the DVM as offering a private and discreet way to access HIVST kits, potentially reducing fear of judgement commonly associated with clinic-based testing.

People stare too much … older people would wonder why a young person would test for HIV … that’s why some people are scared to get tested at the clinic. [Female FGD participant]

Similarly, the healthcare workers were of the view that having AYP discreetly access HIVST kits from the DVM would make them comfortable to test for HIV.

At least there will not be anyone to discriminate against them … no one will see them so they will feel more comfortable. [Healthcare worker, individual interview]

This view suggests that privacy enhances the relative advantage of the DVM by addressing stigma-related barriers, highlighting the importance of anonymity in increasing HIV testing uptake among young people.

#### Faster access to HIV self-test kits

The participants were of the view that a DVM could reduce waiting times associated with facility-based testing by enabling quick access to HIVST kits.

It can be faster unlike those going to queue up, you will just test from home. [Male FGD participant]

Healthcare workers similarly reported that long waiting times discourage young people from accessing HIV services.

Most of the youths … say they are discouraged because when they come in the morning they leave at 12 hours. [Healthcare worker, individual interview]

This means that reduced waiting time may potentially contribute to the perceived efficiency of the DVM, reinforcing its advantage over conventional testing pathways that are often time-consuming.

#### Promotion of routine testing

The healthcare workers and young people were of the view that strategically placing the DVMs in youth-friendly spaces could promote routine HIV testing and broader sexual health awareness.

If it is put in highly populated areas where young people are always found … it will be easier to access testing kits. [Healthcare worker, individual interview]

Young people indicated that proximity could encourage more frequent testing compared to clinic-based services.

Sometimes we stay a year without testing but if it’s close to you, you will keep getting test kits … rather than coming to the clinic. [Female FGD participant]

These perspectives reflect the potential of the DVM to normalize HIV testing and promote routine health-seeking behaviour, particularly among young people who may otherwise delay testing.

## Relative advantage of the mobile phone application

### Timely support and information on linkage to care

The participants perceived the mobile application as enabling quicker access to information on how to interpret the HIV test results and linkage to other supportive services, saving time on visiting the health facility for such information. This was considered beneficial for young people balancing school and family responsibilities.

… I would rather they send them to my phone because you will find a long queue … it’s better you get tested and you go your way. [Female FGD participant]

The application should not just support the results but also a request to see the doctor again. [Male FGD participant]

This view suggests that the app’s perceived advantage lies in reducing delays, particularly for young people balancing competing responsibilities. Its perceived advantage is also contingent on integrating emotional and clinical support mechanisms.

Despite acknowledging the potential benefits of the mobile application, the participants raised concerns about privacy and confidentiality particularly regarding hacking, unauthorized phone access, and limited password protection on shared devices. These concerns reduced the perceived advantage of app-based HIV test results delivery.

… there should be an alert when someone wants to hack this app … [Female FGD participant]

… these small phones have no passwords … when the message arrives, they will begin to talk about you … [Female FGD participant]

The healthcare workers suggested that additional security features to the mobile application could improve acceptability.

… If the app could have features like a password before you see the message coming maybe it will have some privacy … [Peer support worker, individual participant]

These concerns may indicate that the perceived risks to confidentiality could undermine the relative advantage of digital result delivery, emphasizing the need for robust data security features.

## Complexity

### Complexity of using the digital vending machine

#### User interface and navigation

The participants emphasized that the design and interface of the DVM would strongly influence ease of use. Both the AYP and healthcare workers recommended incorporating symbols, coluorful illustrations and voice-over instructions in different local languages to support users with low literacy. Ambiguous instructions or unclear graphics were perceived as increasing the difficulty of independently operating the machine.

It should have option and maybe steps in different languages like Nyanja and the person will choose, just like what airtel has done. [Female FGD participant]

They can put signs, so it is easier for someone who cannot read. [Male FGD participant]

… if it has digits that you press … it will be very easy. [Healthcare worker, individual interview]

Ease of use was considered a key determinant of perceived complexity of the DVM, particularly in settings with varying literacy levels.

#### Familiarity with vending technology

The participants reported that prior exposure to vending machines and automated technologies such as Automated Teller Machines (ATMs) could help overcome any perceived complexity. This familiarity was seen as facilitating confidence in interacting with the DVM and lowering the learning curve.

It is like that big machine where you put a coin, and drinks come out. [Female FGD participant]

It looks like an ATM … it is just a matter of putting it somewhere and educating them. [Peer support worker, individual interview]

This perspective reflects the need for implementers of the DVM to leverage familiar technologies, such as ATMs, to enhance user confidence.

## Complexity of using the mobile phone application

### Literacy and interpretation of results

Low literacy levels were identified as an important factor influencing the perceived complexity of using the mobile app. The AYP expressed concern that users who struggle with reading and writing might find it difficult to understand the information presented within the app. They emphasized the need for clear and simplified language to support comprehension.

… consider those that are unable to read and write … [Female FGD participant]

The healthcare workers also stressed that technical or clinical language could increase difficulty in interpreting results.

… the information should be simple. If it is English, it should not be complicated like the one we use at the hospital that people will not understand out there … [Healthcare worker, individual interview]

The perceived complexity of using the mobile application could be influenced by both digital literacy and clarity of information, underscoring the importance of a simplified design.

## Compatibility

### Compatibility of the digital vending machine

#### Safety and security concerns

Both AYP and healthcare workers expressed concerns about vandalism and theft, which they perceived as issues that could influence the compatibility of the DVM with the community. They noted that past incidents of theft and damage to community infrastructure undermine confidence in the practicality of installing vending machines in open public spaces.

… there are junkies who will steal and get things from the machine …. [Male FGD participant]

Healthcare workers emphasized the need for secure placement of the DVMs to enhance acceptability.

It has to be at a place that is safer because someone might temper with it …. [Healthcare worker, individual interview]

These concerns highlight how community context may limit the perceived compatibility of the DVM.

#### Perceived privacy benefits

Despite security concerns, participants indicated that the DVM aligned with young people’s need for privacy when accessing HIV testing. The anonymous nature of vending machines was seen as compatible with efforts to minimize stigma and embarrassment associated with clinic-based HIV testing.

I also prefer the vending machine because many feel embarrassed to get a test so you can easily press this …. [Female FGD participant]

Healthcare workers similarly reported that stigma remains a barrier to facility-based HIV testing, making the DVM more attractive.

… many do not come to the facility because they are afraid of stigma and being looked down on if she is discovered to be HIV positive. [Healthcare worker, individual interview]

These perspectives show that compatibility of the DVM with the social context may be strengthened by factoring in existing social concerns around stigma.

#### Integration with existing healthcare systems

Healthcare workers raised concerns that integrating DVMs into existing healthcare systems may be challenging and could disrupt established workflows. Traditional HIV testing services are typically embedded within structured health facility processes where testing and counselling and immediate linkage to care occur in a coordinated manner. This lack of integration could lead to gaps in follow-up care, limited data for monitoring and personalized counselling.

Yeah, it will be very ok for them but then health wise for us practitioners it will be like work done is equal to zero. Someone can have the results, and we have not spoken to them then there is nothing that can be done. [Healthcare worker, individual interview]

These perspectives suggest potential misalignment between the DVM with existing care pathways, particularly regarding follow-up care and counselling.

#### Maintenance and technical reliability

Participants also highlighted concerns about ongoing maintenance and potential technical malfunctions. These issues were perceived as affecting the DVM’s compatibility with community expectations for reliable service delivery.

The machine is good, but another thing is maintenance. How will it be maintained? [Male FGD participant]

#### Linkage to care and emotional support

Most young people had concerns about counselling and emotional support when one tests positive and some said that they are not emotionally stoic and could not handle a reactive result without any form of encouragement or emotional support. Consequently, they perceived testing at a health facility as better than going to get tested on their own after accessing the vending machines.

Going to the clinic is better … you need to go to the clinic, get counselled and get medication. [Female FGD participant]

Healthcare workers echoed this concern, noting uncertainty about post-test follow-up.

… they can access the results there … but afterwards what’s going to happen? [Healthcare worker, individual interview]

These perspectives highlight the importance of integrating healthcare worker support within digital interventions.

## Compatibility of the mobile phone application

### Technological accessibility

Participants described smartphone ownership and digital exposure among young people as supporting the application’s compatibility with their daily lives. Where smartphones and connectivity were available, the app was viewed as a natural extension of existing digital practices.

These days every young person has a phone so it will be easy for them … and they are fully aware of technology so it would be easy to use it. [Peer support worker, individual interview]

These views suggest that deployment of the mobile application may have to consider the existing digital behaviours of AYP to enhance the application’s compatibility with users’ daily lives.

### Familiarity with mobile technology

Young people’s routine use of mobile applications for communication and entertainment further reinforced perceptions of compatibility. Participants noted that digital platforms are already embedded in young people’s social interactions.

… everyone will find it better because of technology and since many have phones …. [Male FGD participant]

### Internet cost considerations

Both AYP and healthcare workers perceived the need for mobile data bundles as a potential barrier to compatibility. Participants emphasized that data costs could restrict consistent use of the application but noted the app did not require significant data to use.

… this app should be free because it has no pictures …. [Male FGD participant]

… if it will need bundles then it will be very challenging. [Healthcare worker, individual interview]

This suggests that economic and infrastructural barriers may potentially reduce compatibility of the mobile app for some users. Therefore, deployment of the app may require that implementors consider the cost implications of effectively using it.

## Discussion

This study sought to explore the appropriateness of DVMs and a mobile phone application in increasing access to HIV testing among AYP aged 18–24 in an Urban Community, in Lusaka district. Overall, our findings reflect participants’ views of a proposed digital HIV testing pathway rather than actual experiences of use. However, given that this work was part of the formative research conducted during the co-design process of a digitally supported care pathway to facilitate access of comprehensive sexual reproductive health services to AYP, we believe that our findings are valuable for those developing and planning to deploy similar interventions in comparable contexts.

The relative advantage of accessing HIVST kits from the DVMs compared to existing facility-based services was linked to their perceived accessibility across various community locations without the need to visit a health facility during specific hours. These locations may include sports centres, schools, night clubs and youth hubs which exist within the community. Further, participants perceived the machines as promoting privacy and anonymity when acquiring HIV test kits. This perceived anonymity may, however, be challenged by the placement of DVM in public, high-traffic locations, where being seen accessing the machine could expose AYP to social scrutiny, highlighting a potential tension between accessibility and privacy that should be carefully considered during deployment. In contrast, a study in Japan found that the uptake of HIV self-testing distributed via DVM was driven by convenience and time savings rather than privacy concerns [23]. This highlights how the perceived relative advantage of digital interventions may vary across contexts depending on underlying social norms and stigma related to HIV testing. A study conducted in the United Kingdom also found that young people perceived digitally supported pathways as advantageous because they reduced the need for potentially uncomfortable face-to-face interactions in health facilities [24].

Receiving support for HIV test results through a mobile phone application was perceived as advantageous compared to waiting for test results and advice at health facilities, particularly due to faster access to self-test results. Participants’ views were shaped by experiences of long waiting times at the local health facility, where high patient volumes and limited staffing may delay service delivery. However, this perceived benefit was likely context-dependent and may not be transferable to settings with more efficient service delivery systems. Long waiting times tend to discourage AYP from completing the HIV testing process and this is shared with their colleagues, affecting their willingness to test. Therefore, participants felt that a mobile application may help reduce the waiting times at facilities and complement use of DVM. Similarly, a study conducted in the UK found that participants preferred mobile-based HIV services due to their potential to bring information about a health intervention together in one connected pathway [24].

Participants also perceived mobile phone-based support as convenient, given the widespread ownership and daily use of mobile phones among young people. This aligns with findings from a study conducted in Lusaka by Bukowa [25], which identified mobile phones as the most commonly used devices for accessing the internet and social media among young people. Evidence from Uganda further suggests that adolescents are open to mobile phone-based HIV interventions, with a substantial proportion expressing willingness to engage with such platforms [26]. However, these perceptions reflect anticipated benefits rather than actual usage behaviours and further research is needed to assess real-world feasibility and engagement.

Familiarity with digital technologies, such as ATMs and mobile applications, was seen as enhancing compatibility of the proposed DVM and mobile application interventions. Participants noted that AYP are generally attracted to novel and technology-driven approaches, suggesting that integrating HIV testing into such platforms may increase uptake. Similar findings were reported in a study conducted in the USA, where DVM were perceived as acceptable due to their novelty and convenience [14]. Alignment of new innovations with prevailing practices or interests in the community is important because it reduces possible conflicts among the adopters which can either delay or distort the acceptability process of the new intervention [27–29]. Such alignments appear to have provided digital vending machines and mobile phone applications with a clear advantage over the existing health programs or approaches, a situation which according to the DOI theory, significantly influences the rate and pattern of adoption [30,31].

Meanwhile, low literacy levels in the community were identified by both AYP and healthcare workers as a key barrier to using digital health technologies. Participants expressed concerns that complex interfaces or unclear instructions could discourage use, leading to abandonment of the intervention. This suggests that perceived complexity may negatively influence adoption. Evidence from Bangladesh indicates that mHealth interventions targeting low-literacy populations are more effective when incorporating voice instructions, visuals and simplified navigation [32]. Similarly, a review by Aljedaani and Babar highlights the importance of user-centred design in improving usability and outcomes in low-resource settings [33]. A study in Japan also emphasized that simple and visually appealing vending machine designs contributed to increased uptake of HIV testing [23].

## Strength and limitations

This study benefited from the inclusion of multiple participant groups, including AYP, healthcare workers and peer support workers which provided diverse perspectives on the proposed digital HIV testing Pathway. Another strength of our study was iterative coding process used by the research team that ensured a consensus-led approach to naming, organizing and reporting of the themes. Furthermore, the use of both FGDs and IDIs enhanced the depth and credibility of findings through triangulation. However, our study had several limitations. First, we only included AYP who had previously undergone HIV testing. Therefore, our findings may not fully reflect the perspectives of young people who have never been tested for HIV. Further, perceptions regarding relative advantage, compatibility and complexity were based on anticipated rather than actual experiences with the technologies hence our findings do not provide evidence on real-world uptake. Our findings should therefore be interpreted with caution as participants’ views may differ from experiences that emerge during actual use. Although we operationalized appropriateness through the DOI constructs, appropriateness may encompass broader dimensions beyond innovation attributes, including contextual, organizational and implementation-related factors. Lastly, the study participants did not provide feedback on the findings following the data analysis.

## Conclusion

Findings from this study provide insights into the perceived appropriateness of using DVM and mobile phone applications in increasing access to HIV testing for young people. Perceived advantages, such as convenience, privacy and ease of access to results, suggest that these interventions may be appropriate within this population. Concerns related to privacy in public settings, digital literacy and access to counselling highlight important considerations for implementation.

While these findings indicate potential to improve access to HIV testing, they should not be interpreted as evidence of effectiveness as the study did not assess actual use, uptake or health outcomes. Further research should focus on pilot implementation and evaluation of these interventions in real-world settings to better understand their feasibility, acceptability and impact.

## Supplementary Material

Sarah Mukimba_ISSM_COREQ_Checklist .pdf

## Data Availability

The dataset utilized and analysed in this study are accessible and can be obtained from the corresponding author, Sarah Mukimba, upon making a reasonable request.

## Preprint

This manuscript has not been previously published on any preprint server.

## References

[1] Sokhan A. Global, regional, and national burden of HIV/AIDS, 1990–2021, and forecasts to 2050, for 204 countries and territories: the Global burden of disease study 2021. Lancet HIV. 2024;11:e807–12. doi: 10.1016/S2352-3018(24)00212-1 PMC11612058 39608393

[2] Zhang J, Ma B, Han X, et al. Global, regional, and national burdens of HIV and other sexually transmitted infections in adolescents and young adults aged 10–24 years from 1990 to 2019: a trend analysis based on the Global burden of disease study 2019. The Lancet Child Adolesc Health. 2022;6:763–776. doi: 10.1016/S2352-4642(22)00219-X 36108664

[3] Zambia Stastical Agency. Zambia Population-Based HIV Impact Assessment Final Report. 2023; Available from: https://www.google.com/url?sa=t&source=web&rct=j&opi=89978449&url=https://www.zamstats.gov.zm/wp-content/uploads/2024/01/ZAMPHIA-2021-Final-Report.pdf&ved=2ahUKEwj-3PvHstmTAxXjQ6QEHX3QBHIQFnoECBsQAQ&usg=AOvVaw16YYtnhkEMxCN6UznzoUI9

[4] National AIDS Council of Zambia. National HIV & AIDS Strategic Framework. 2023–2027. Available from: https://nac.org.zm/download/national-aids-strategic-framework-nasf-2023-2027/#

[5] Frescura L, Godfrey-Faussett P, Feizzadeh A, et al. Achieving the 95 95 95 targets for all: a pathway to ending AIDS. In: Zandrea A, editor. Vol. 17. PLoS One. 2022. p. e0272405. doi: 10.1371/journal.pone.0272405 PMC9352102 35925943

[6] Mwaba K, Mannell J, Burgess R, et al. Uptake of HIV testing among 15–19-year-old adolescents in Zambia. AIDS Care. 2020;32:183–192. doi: 10.1080/09540121.2020.1739214 32169008

[7] Organization WH. Adolescent friendly health services for adolescents living with HIV: from theory to practice. Dec 2019: technical Brief. 2019;5–16.

[8] Hensen B, Sian F, Phiri MM, et al. The impact of community-based, peer-led sexual and reproductive health services on knowledge of HIV status among adolescents and young people aged 15 to 24 in Lusaka, Zambia: the Yathu Yathu cluster-randomised trial. PLoS Med. 2023;20:e1004203. doi: 10.1371/journal.pmed.1004203 PMC10121029 37083700

[9] Zanolini A, Chipunga J, Vinikoor MJ, et al. HIV self-testing in Lusaka Province, Zambia: acceptability, comprehension of testing instructions, and individual preferences for self-test kit distribution in a population-based sample of adolescents and adults. AIDS Res Hum Retroviruses. 2018;34:254–260. doi: 10.1089/aid.2017.0156 PMC5863088 28969432

[10] Indravudh PP, Sibanda EL, d’Elbée M, et al. ‘I will choose when to test, where I want to test’: investigating young people’s preferences for HIV self-testing in Malawi and Zimbabwe. AIDS. 2017;31:S203–S212. doi: 10.1097/QAD.0000000000001516 PMC5497773 28665878

[11] Nwaozuru U, Iwelunmor J, Ong JJ, et al. Preferences for HIV testing services among young people in Nigeria. BMC Health Serv Res. 2019;19:1003. doi: 10.1186/s12913-019-4847-x PMC6935128 31881959

[12] Vera JH, Soni S, Pollard A, et al. Acceptability and feasibility of using digital vending machines to deliver HIV self-tests to men who have sex with men. Sex Transm Infect. 2019;95:557–561. doi: 10.1136/sextrans-2018-053857 31101723

[13] Neuman M, Mwinga A, Kapaku K, et al. Sensitivity and specificity of OraQuick® HIV self-test compared to a 4th generation laboratory reference standard algorithm in urban and rural Zambia. BMC Infect Dis. 2022;22:494. doi: 10.1186/s12879-022-07457-5 PMC9134574 35614397

[14] Young SD, Daniels J, Chiu CJ, et al. Acceptability of using electronic vending machines to deliver oral rapid HIV self-testing kits: a qualitative study. PLoS One. 2014;9:e103790. doi: 10.1371/journal.pone.0103790 PMC4116256 25076208

[15] Stafylis C, Natoli L, Murkey J, et al. Vending machines in commercial sex venues to increase HIV self-testing among men who have sex with men. Mhealth. 2018;4:51. doi: 10.21037/mhealth.2018.10.03 PMC6232061 30505849

[16] Proctor E, Silmere H, Raghavan R, et al. Outcomes for implementation research: conceptual distinctions, measurement challenges, and research agenda. Adm Policy Ment Health Ment Health Serv Res. 2011;38:65–76. doi: 10.1007/s10488-010-0319-7 PMC3068522 20957426

[17] Dearing JW. Applying diffusion of innovation theory to intervention development. Res Soc Work Pract. 2009;19:503–518. doi: 10.1177/1049731509335569 PMC2957672 20976022

[18] Dearing JW, Cox JG. Diffusion of innovations theory, principles, and practice. Health Aff. 2018;37:183–190. doi: 10.1377/hlthaff.2017.1104 29401011

[19] Tong A, Sainsbury P, Craig J. Consolidated criteria for reporting qualitative research (COREQ): a 32-item checklist for interviews and focus groups. Int J Qual Health Care. 2007;19:349–357. doi: 10.1093/intqhc/mzm042 17872937

[20] Zambia Stastical Agency. Census of Population and Housing. 2022; Available from: https://www.zamstats.gov.zm/zamstats-to-release-more-census-reports-january-2025/

[21] Braun V, Clarke V. Using thematic analysis in psychology. Qualitative Res Phychol. 2006;3:77–101. doi: 10.1191/1478088706qp063oa

[22] Williams JR. The declaration of Helsinki and public health. Bull World Health Organ. 2008;86:650–652. doi: 10.2471/BLT.08.050955 PMC2649471 18797627

[23] Kaneko N, Sherriff N, Takaku M, et al. Increasing access to HIV testing for men who have sex with men in Japan using digital vending machine technology. Int J STD AIDS. 2022;33:680–686. doi: 10.1177/09564624221094965 PMC9189596 35502984

[24] Aicken CR, Fuller SS, Sutcliffe LJ, et al. Young people’s perceptions of smartphone-enabled self-testing and online care for sexually transmitted infections: qualitative interview study. BMC Public Health. 2016;16:974. doi: 10.1186/s12889-016-3648-y PMC5022229 27624633

[25] Bukowa B. Young people and the digital age: an investigation of the ‘Sociability’of teenagers in Lusaka. 2018; Available from: https://dspace.unza.zm/server/api/core/bitstreams/b309e092-6045-47b5-94ff-0f943a6def3c/content

[26] Kreniske P, Basmajian A, Nakyanjo N, et al. The promise and peril of mobile phones for youth in rural Uganda: multimethod study of implications for health and HIV. J Med Internet Res. 2021;23:e17837. doi: 10.2196/17837 PMC7886611 33528375

[27] Atun R, de Jongh T, Secci F, et al. Integration of targeted health interventions into health systems: a conceptual framework for analysis. Health Policy Plan. 2010;25:104–111. doi: 10.1093/heapol/czp055 19917651

[28] Getachew A, Silumbwe A, Maritim P, et al. An evaluation of community acceptability and adoption of the community-based TB care program in Ethiopia using the diffusion of innovation model. J Community Syst Health. 2024;1. doi: 10.36368/jcsh.v1i1.1072

[29] Noella KK, Silumbwe A, Munakampe NM, et al. Acceptability and adoption of integrated malaria case management at community level in under-five children in Manono health zone, democratic republic of Congo. J Community Syst Health. 2026;3. doi: 10.36368/jcsh.v3i1.1255

[30] Banda E, Svanemyr J, Sandoy I, et al. Acceptability of an economic support component to reduce early pregnancy and school dropout in Zambia: a qualitative case study. Glob Health Action. 2019;12:1685808. doi: 10.1080/16549716.2019.1685808 PMC6882474 31735121

[31] Zulu JM, Hurtig A-K, Kinsman J, et al. Innovation in health service delivery: integrating community health assistants into the health system at district level in Zambia. BMC Health Serv Res. 2015;15:38. doi: 10.1186/s12913-015-0696-4 PMC4314770 25627456

[32] Islam MN, Karim MM, Inan TT, et al. Investigating usability of mobile health applications in Bangladesh. BMC medical informatics and decision making. BMC Med Inf Decis Mak. 2020;20:19. doi: 10.1186/s12911-020-1033-3 PMC6998368 32013965

[33] Aljedaani B, Babar MA. Challenges with developing secure mobile health applications: systematic review. JMIR Mhealth Uhealth. 2021;9:e15654. doi: 10.2196/15654 PMC8277314 34152277

